# Reply to Kuo et al. Comment on “Du et al. Gender Differences in the Relationships between Perceived Stress, Eating Behaviors, Sleep, Dietary Risk, and Body Mass Index. *Nutrients* 2022, *14*, 1045”

**DOI:** 10.3390/nu14193882

**Published:** 2022-09-20

**Authors:** Chen Du, Mary Adjepong, Megan Chong Hueh Zan, Min Jung Cho, Jenifer I. Fenton, Pao Ying Hsiao, Laura Keaver, Heesoon Lee, Mary-Jon Ludy, Wan Shen, Winnie Chee Siew Swee, Jyothi Thrivikraman, Felicity Amoah-Agyei, Emilie de Kanter, Wenyan Wang, Robin M. Tucker

**Affiliations:** 1 Department of Food Science and Human Nutrition, Michigan State University, East Lansing, MI 48824, USA; 2 Department of Biochemistry, Kwame Nkrumah University of Science and Technology, Kumasi 00233, Ghana; 3 Division of Nutrition and Dietetics, International Medical University, Kuala Lumpur 57000, Malaysia; 4 Global Public Health, Leiden University College, 2595 DG The Hague, The Netherlands; 5 Department of Food and Nutrition, Indiana University of Pennsylvania, Indiana, PA 15705, USA; 6 Department of Health and Nutritional Science, Institute of Technology Sligo, F91 YW50 Sligo, Ireland; 7 Department of Human Services, Bowling Green State University, Bowling Green, OH 43403, USA; 8 Department of Public and Allied Health, Bowling Green State University, Bowling Green, OH 43403, USA

We thank Kuo et al. for their comments [1] on our paper [2] entitled “Gender differences in the relationships between perceived stress, eating behaviors, sleep, dietary risk, and body mass index”. They remind us that there are different statistical approaches to exploring mediating relationships between variables, and that researchers should carefully consider which methods are best for their analyses.

Mediation analysis is a dynamic and evolving field. Kuo et al. raise the use of the causal steps approach, which was developed in the 1980s [3]. In our paper, we used the more recently developed product of coefficients approach [4,5,6,7]. We selected the product of coefficients approach with resampling (bootstrapping) as this method addresses several limitations of the causal steps method, for instance: (1) low power to detect a mediation effect, which translates to requiring large sample sizes and makes studies impossible in many cases [8]; (2) the power to detect mediation effects using the causal steps approach approximately equals the power to detect mediation by testing whether the relationship between an independent variable and a mediator and the relationship between a mediator and a dependent variable are significant [4]; (3) many studies demonstrate that a significant mediation effect exists in the absence of a significant relationship between an independent and dependent variable [3,8,9]; (4) the causal steps approach does not employ resampling [4], which can lead to the issue of random effects causing sampling bias [10]. Given these limitations of the causal steps methodology, we decided to use the product of coefficients with resampling approach.

To address the validity, we based the models on sound theoretical frameworks from the available literature [4,9,11]. Additionally, we adjusted covariates based on previous work, including age, country, citizenship status, and class status [12,13,14,15]. In terms of sleep duration and quality, these variables were included in the models as moderators based on the literature [16,17,18,19,20,21]; therefore, they were not included in the models as covariates. Given our decision to minimize the presence of independent-variable-induced mediator-outcome confounders by developing models based on previous findings and adjusted for covariates, sensitivity analysis of the indirect effect was not performed [22].

We agree with Kuo et al. that students should be trained in multiple methods of mediation analyses in order to make informed decisions about the optimal approach for analyzing their data.
